# New insights from uncultivated genomes of the global human gut microbiome

**DOI:** 10.1038/s41586-019-1058-x

**Published:** 2019-03-13

**Authors:** Stephen Nayfach, Zhou Jason Shi, Rekha Seshadri, Katherine S. Pollard, Nikos C. Kyrpides

**Affiliations:** 1 United States Department of Energy Joint Genome Institute, Walnut Creek, CA USA; 2 Environmental Genomics and Systems Biology Division, Lawrence Berkeley National Laboratory, Berkeley, CA USA; 3 Gladstone Institutes, San Francisco, CA USA; 4 Chan-Zuckerberg Biohub, San Francisco, CA USA; 5 Institute for Human Genetics, University of California San Francisco, San Francisco, CA USA; 6 Institute for Computational Health Sciences, University of California San Francisco, San Francisco, CA USA; 7 Quantitative Biology Institute, University of California San Francisco, San Francisco, CA USA; 8 Department of Epidemiology and Biostatistics, University of California San Francisco, San Francisco, CA USA

**Keywords:** Environmental microbiology, Genome informatics

## Abstract

The genome sequences of many species of the human gut microbiome remain unknown, largely owing to challenges in cultivating microorganisms under laboratory conditions. Here we address this problem by reconstructing 60,664 draft prokaryotic genomes from 3,810 faecal metagenomes, from geographically and phenotypically diverse humans. These genomes provide reference points for 2,058 newly identified species-level operational taxonomic units (OTUs), which represents a 50% increase over the previously known phylogenetic diversity of sequenced gut bacteria. On average, the newly identified OTUs comprise 33% of richness and 28% of species abundance per individual, and are enriched in humans from rural populations. A meta-analysis of clinical gut-microbiome studies pinpointed numerous disease associations for the newly identified OTUs, which have the potential to improve predictive models. Finally, our analysis revealed that uncultured gut species have undergone genome reduction that has resulted in the loss of certain biosynthetic pathways, which may offer clues for improving cultivation strategies in the future.

## Abstract (web summary)

Draft prokaryotic genomes from faecal metagenomes of diverse human populations enrich our understanding of the human gut microbiome by identifying over two thousand new species-level taxa that have numerous disease associations.

## Main

The gut microbiome has myriad important roles in human health and disease^1^. Microbial reference genomes are essential resources for understanding the functional role of specific organisms in the microbiome, and for quantifying their abundance from metagenomes^2^. However, an estimated 40–50% of human gut species lack a reference genome^3,4^. Although considerable efforts have been made to culture and sequence members of the gut microbiome^5–7^, many microorganisms have not been grown under laboratory conditions to date and lack a sequenced genome—despite being prevalent in humans^8^.

Recent advances in experimental technologies have begun to close this gap: some studies^6,7^ have used microbial culturomics to isolate and sequence hundreds of previously uncultured organisms in the human gut, and others have performed single-cell genome sequencing^9^. In contrast to experimental approaches, metagenome binning is a computational approach that can be used to obtain genomes directly from samples without isolation or culturing. Sequencing reads are first assembled into contigs, which are then binned into metagenome-assembled genomes (MAGs) on the basis of nucleotide frequency, abundance and/or co-variation of abundance across a group of samples^10^. This process is performed either for individual metagenomes^11^ or multiple co-assembled metagenomes^12^. MAGs are subsequently evaluated for various indicators of genome quality, including estimated completeness and contamination, the presence of marker genes and overall contiguity^13–15^.

MAGs were first assembled from a low-complexity acid-mine drainage community^16^ but—with advances in sequencing technology and computational methods—MAGs have now been recovered from a myriad of environments including the global ocean^17^, cow rumen^12^, aquifer systems^18^ and others^11^. These uncultured genomes have expanded the tree of life by revealing novel lineages in diverse environments, as well as unusual biology^11,19^. Despite the growing number of publicly available human gut metagenomes, there has not been any large-scale assembly of MAGs from the gut microbiome. Nielsen et al.^20^ were the first to recover MAGs from gut metagenomes, and similar concepts have been developed and applied to other individual studies^21^. We hypothesized that human gut MAGs systematically recovered from public metagenomes could substantially increase the diversity of species with a sequenced genome, and shed light on the biology of uncultivated organisms in the gut microbiome.

## Reconstructing genomes from global gut metagenomes

**Fig. 1:**
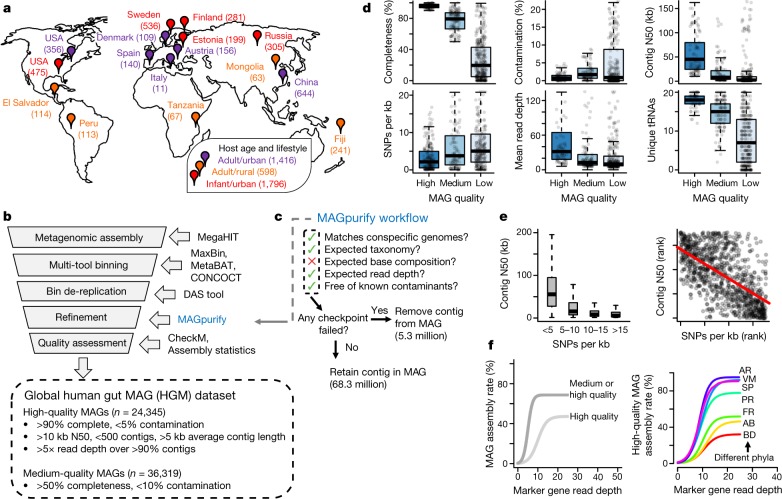
Recovery of genomes from globally distributed gut metagenomes. **a**, Geographical distribution of metagenomes. Sample sizes are indicated in parentheses, and pin colour indicates the majority age group and lifestyle (infants, ≤3 years old; adults, ≥18 years old). Several locations are represented by multiple studies; several studies were conducted in multiple locations. **b**, Computational pipeline for assembling MAGs. **c**, Pipeline for identifying and removing incorrectly binned contigs. **d**, Quality metrics across low- (*n* = 101,651), medium- (med., *n* = 36,319) and high-quality (*n* = 24,345) MAGs. **e**, Barriers to MAG recovery. Single nucleotide polymorphisms (SNPs) were called for MAGs with sufficient read depth (*n* = 17,671), and compared with N50. Red line is from a Spearman correlation (*ρ* = −0.61). **f**, At least 10–20× depth is required to assemble a MAG, but assembly rates vary between taxa. AB, Actinobacteria; AR, Archaea; BD, Bacteroidetes; FR, Firmicutes; VM, Verrucomicrobia; PR, Proteobacteria; SP, Spirochaetes. Sequencing read depth was estimated using IGGsearch (see Methods), and curves were fit using logistic regression. For box plots, the middle line denotes the median; the box denotes the interquartile range (IQR); and the whiskers denote 1.5× IQR.

**Extended Data Fig. 1:**
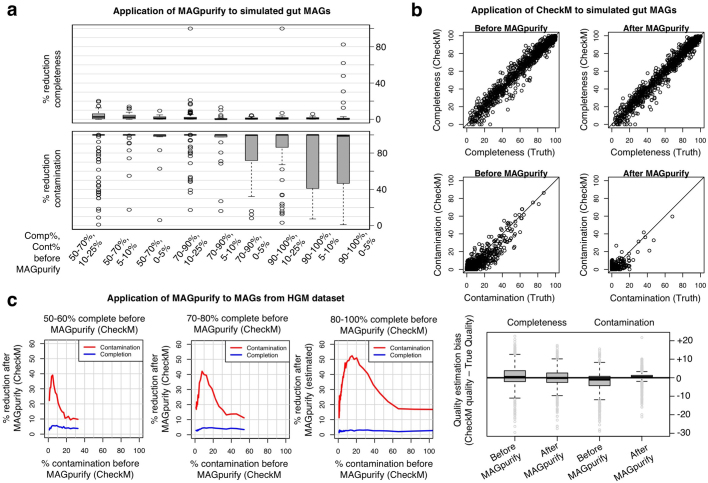
The MAGpurify tool removes contamination, maintains completeness and does not result in biased estimates of genome quality. **a**, **b**, One thousand human gut MAGs were simulated to validate the MAGpurify pipeline. Each MAG contained two genomes: one host genome that represents the target genome, and one donor genome that represents the contaminating genome (Supplementary Table 7). All 102 input genomes were isolated from the human gut, and were estimated to have >95% completeness, <1% contamination and <25 contigs. MAGs were simulated with completeness, contamination and N50 on the basis of randomly sampled MAGs from the HGM dataset. Sixty-five MAGs in which contamination exceeded completeness (and thus the host genome was in the minority) were dropped from the analysis. **a**, The box plots indicate the percentage of reduction in completeness (top) and contamination (bottom) after applying MAGpurify. Regardless of initial quality, MAGpurify sensitively removed contamination for most MAGs, while avoiding removal of the host genome. **b**, CheckM was applied to simulated MAGs before and after applying MAGpurify. Top, the scatter plots show that true genome quality is correlated with the estimated genome quality before and after applying MAGpurify. Black lines indicate the line of equality. Bottom, the distribution of differences between true and estimated quality is centred at zero, which indicates that CheckM quality estimates are not biased after applying MAGpurify. **c**, MAGpurify was applied to all MAGs from the HGM dataset. The figure shows the reduction in CheckM quality estimates before and applying MAGpurify. Estimated quality improvement is greatest when completeness is between 90 and 100% and contamination is between 10 and 30%. In all box plots, the middle line denotes the median, the box denotes the IQR and the whiskers denote 1.5× IQR.

**Extended Data Fig. 2:**
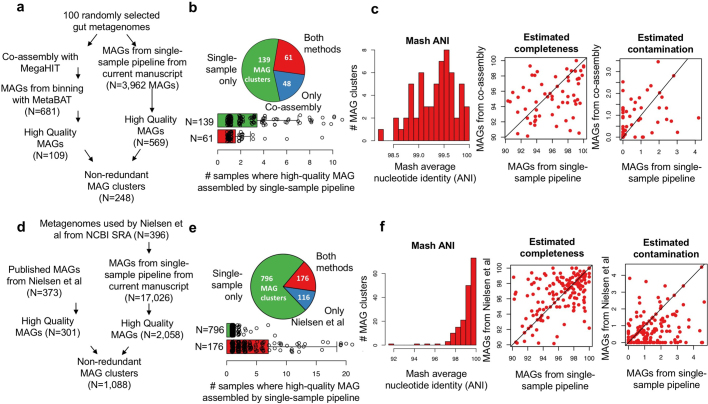
Single-sample assembly and binning yields more non-redundant, high-quality MAGs compared to other approaches. **a**–**c**, Comparison of single-sample assembly and binning with co-assembly and binning. **a**, One hundred randomly selected human gut metagenomes were co-assembled with MegaHIT (v.1.1.4, options ‘–k-min 27–k-max 127–k-step 10–kmin-1pass–continue’), which took 3,608 central processing unit hours. Reads from each sample were mapped back to the co-assembly to quantify the read depth of each contig in each sample. This information was used as input to MetaBAT (v.2.12.1, default options) to generate MAGs. Other binning programs—including CONCOCT and MaxBin2—did not complete owing to the large size of the assembly. MAGs from the single-sample pipeline were grouped with MAGs from the co-assembly using Mash at 90% ANI to form 248 clusters. **b**, A large fraction of clusters is exclusively represented by MAGs from the single-sample pipeline. These clusters tend to be found in multiple samples, which may interfere with co-assembly. For bar plots, the centre bar indicates the mean, the error bar indicates the standard deviation and all data points are overlaid. **c**, The MAGs recovered by both pipelines (*n* = 61) have high ANI (which indicates that they are very similar genomes) and tend to have similar levels of estimated completeness and contamination, as determined by CheckM. Black lines indicate the line of equality. **d**–**f**, Comparison of single-sample assembly and binning with co-abundance binning (as previously performed^20^). **d**, MAGs from the single-sample pipeline were grouped with previously published MAGs^20^ using Mash at 90% ANI to form 1,088 clusters. **e**, A large fraction of clusters is only represented by MAGs from the single-sample pipeline, which tend to be restricted to individual metagenomes—this may be explained by the fact the previously published^20^ method requires MAGs to be present in multiple samples to accurately quantify co-variation and bin contigs. For bar plots, the centre bar indicates the mean, the error bar indicates the standard deviation and all data points are overlaid. **f**, The MAGs recovered by both pipelines (*n* = 176) have high ANI (which indicates that they are very similar genomes) and tend to have similar levels of estimated completeness and contamination, as determined by CheckM. Black lines indicate the line of equality.

To recover genomes for novel human gut lineages, we performed metagenomic assembly and binning on 3,810 globally distributed samples from phenotypically and demographically diverse human subjects, using a pipeline developed for this study (Fig. 1a, b, Supplementary Tables 1–5). MAG quality was improved further by using a pipeline that we developed to identify and remove incorrectly binned contigs (Fig. 1c, Extended Data Fig. 1, Supplementary Tables 6, 7, Methods). We performed single-sample assembly and binning (rather than co-assembly) to preserve strain variation between human hosts, and because co-assembly was not computationally feasible for our large dataset. On the basis of a subset of samples, our pipeline produced 1.8× more non-redundant high-quality MAGs compared to co-assembly, and 3.3× more than a previous study^20^ that used abundance co-variation across samples (Extended Data Fig. 2).

**Extended Data Fig. 3:**
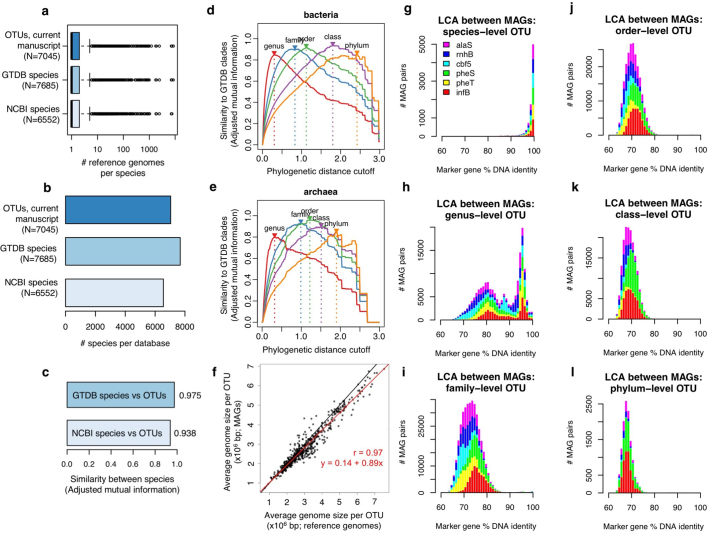
Additional checks of MAG quality after clustering genomes into OTUs. **a**–**c**, MAGs and reference genomes were clustered into species-level OTUs on the basis of 95% ANI. As validation, OTUs were compared to the NCBI and GTDB for 65,900 reference genomes with valid species names. **a**, Box plots of the number of genomes per species, in which the middle line denotes the median, the box denotes the IQR and the whiskers denote 1.5× IQR. **b**, The number of species per database. **c**, Similarity between OTUs and other databases, as measured using the adjusted mutual information statistic. Species-level OTUs are concordant with the NCBI and GTDB taxonomies. **d**, **e**, MAGs and reference genomes were further clustered into higher-rank OTUs on the basis of phylogenetic distance cut-offs. Rank-specific cut-offs were identified that maximized similarity to the GTDB. **f**, As an additional indicator of completeness, genome sizes of high-quality MAGs and reference genomes from the same OTU were compared. Each point indicates one species-level OTU (*n* = 625). A positive slope of close to 1.0 indicates to systematic loss of gene content. **g**–**l**, As an additional check of contamination, six single-copy marker genes (*alaS*, *rnhB*, *cbf5*, *pheS*, *pheT* and *infB*) were aligned between MAGs using BLASTN. MAGs devoid of contamination should display high percentage identity from the same OTU, and low percentage identity between different OTUs. The six marker genes were selected on the basis of (1) their presence in >90% of high-quality MAGs and reference genomes at single copy, and (2) having species-level percentage DNA identity cut-offs <98%. Highly conserved genes may be similar between different OTUs, and were not suitable for this analysis. For between-OTU comparisons we used 1 MAG for each of 2,962 species-level OTUs. For within-OTU comparisons, we used 2 MAGs for each of 1,616 species-level OTUs. The histograms indicate the distribution of DNA percentage identity between MAGs from the same species-level OTU (in which the lowest common ancestor (LCA) = species) (**g**), and between MAGs that are more distantly related, in which the LCA = genus (**h**), family (**i**), order (**j**), class (**k**) or phylum (**l**). The vast majority of genes from the same species-level OTU display >98% identity, whereas those from different OTUs display <98% identity.

Our pipeline yielded 60,664 MAGs that met or exceeded the medium-quality ‘Minimum information about a metagenome-assembled genome’ (MIMAG) standard^14^ which we refer to as the global human gut MAG (HGM) dataset (Fig. 1b, Supplementary Table 8). The MAGs form 43,737 clusters at an average-nucleotide-identity threshold of 99%, which indicates that most of the MAGs are unique. The vast majority of MAGs displayed >98% DNA identity within the same species and <98% identity between species at individual marker genes, which suggests that they are not chimeric (Extended Data Fig. 3g–l). A subset of 24,345 high-quality MAGs was estimated to be near-complete; MAGs in this subset had minimal contamination, high contiguity and were of a similar length to isolate genomes of the same species (Fig. 1b, d, Extended Data Fig. 3f). Only 14.5% of these MAGs were classified as being of high quality by the MIMAG standard, largely owing to the absence of a full complement of rRNA genes—these are challenging to assemble from metagenomes^22^ and are often absent from otherwise near-complete MAGs^11^.

**Extended Data Fig. 4:**
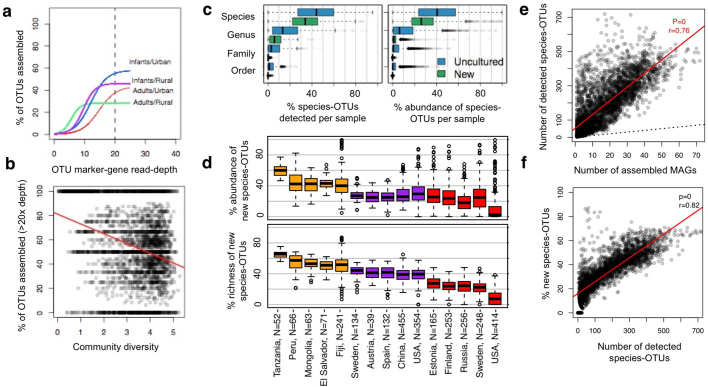
Assembly and distribution of MAGs across human populations. IGGsearch was applied to 3,083 metagenomes from healthy individuals that were used for assembly and binning to estimate the abundance of human gut OTUs per sample. **a**, **b**, The overall assembly rate was computed at each read depth, defined as the percentage of detected OTUs with an assembled MAG. **a**, Curves were fit using logistic regression. Conditioning on read depth, MAGs are recovered more readily from an infant metagenome compared to an adult metagenome from a rural population. **b**, The *x* axis indicates the Shannon diversity of each of the 3,810 metagenomic samples, and the *y* axis indicates the MAG recovery rate for OTUs with >20× depth. MAGs are recovered less often from a high-diversity community, even when read-depth is sufficiently high (Pearson’s *ρ* = −0.31, *P* = 4.3 × 10^−75^). **c**, Relative abundance and richness of newly identified and uncultured OTUs at different taxonomic ranks across metagenomes from healthy individuals (*n* = 3,083). **d**, Data from **c**, but shown only for newly identified species-level OTUs and conditioned by host population. Only populations with at least 30 metagenomes are shown. Orange box plots indicate samples from adults in rural countries, purple from adults in urban countries and red from infants in urban countries. **c**, **d**, In box plots, the middle line denotes the median, the box denotes the IQR and the whiskers denote 1.5× IQR. **e**, IGGsearch sensitively detects the presence of species-level OTUs in samples from which no MAG was recovered. The *x* axis indicated the number of MAGs assembled and the *y* axis indicates the number of species-levels OTUs detected from IGGsearch profiling. Each point indicates one metagenomic sample (*n* = 3,083). The red regression line is from a Pearson correlation. The vast majority of detected species is not assembled into a MAG. **f**, Species richness versus the relative percentage of newly identified species-level OTUs across metagenomic samples (*n* = 3,083). The red regression line is from a Pearson correlation (*ρ* = 0.82, *P* = 0). Newly identified species-level OTUs comprise a greater percentage of the community when diversity is high. This pattern was robust after rarefying metagenomes to one million reads and using a prevalence-matched set of 1,000 newly identified species and 1,000 known species (*ρ* = 0.59, *P* = 0).

Despite the large number of recovered genomes, we identified several challenges to recovering MAGs from human gut metagenomes. First, by mapping reads back to each MAG and quantifying single nucleotide polymorphisms, we confirmed that strain diversity results in highly fragmented MAGs^15^ (Fig. 1e). Second, we found that reliably assembling a MAG required at least 10–20× read depth (Fig. 1f), which indicates that MAGs were only assembled for the most-abundant taxa in each community^23^. MAG assembly was particularly challenging for some phyla—such as Bacteroidetes (Fig. 1f)—and for metagenomes with high community diversity (Extended Data Fig. 4a, b). Despite these challenges, our results indicate that thousands of partial and near-complete genomes can be reconstructed from individual human gut metagenomes using standard pipelines for assembly and binning.

## MAGs represent thousands of unknown species

**Fig. 2:**
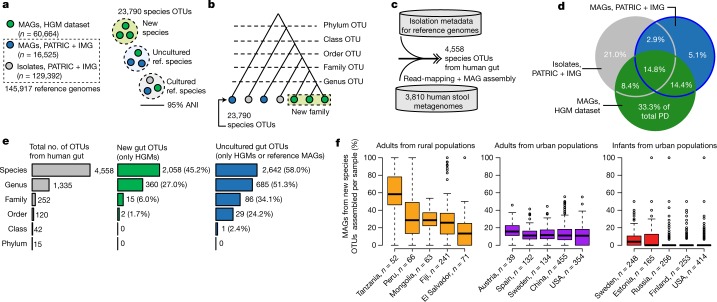
Human gut MAGs expand the genomic diversity of the gut microbiome. **a**, Reference genomes were clustered with MAGs at 95% average nucleotide identity (ANI). IMG, Integrated Microbial Genomes; PATRIC, Pathosystems Resource Integration Center. **b**, All OTUs were further clustered into groups at higher taxonomic ranks. **c**, Human gut OTUs were identified on the basis of isolation metadata, read-mapping or assembly of a gut MAG. **d**, Pie chart indicating the percentage of bacterial phylogenetic diversity (PD) in the gut covered by different sets of genomes. **e**, A considerable fraction of gut OTUs are represented exclusively by MAGs. **f**, Distribution of newly identified OTUs across healthy human populations. Only countries with at least 20 samples are shown. For box plots, the middle line denotes the median; the box denotes the IQR; and the whiskers denote 1.5× IQR.

**Extended Data Fig. 5:**
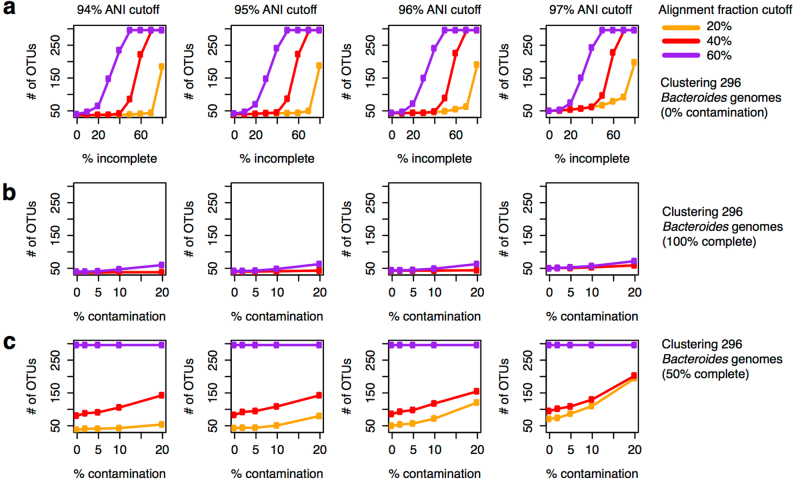
Effect of completeness and contamination on the identification of OTUs from whole genomes. **a**–**c**, OTUs were identified for 296 genomes from the *Bacteroides* genus on the basis of average-linkage clustering of whole-genome ANI, using the ANIcalculator (v.1.0). The ANI cut-offs used for forming OTUs are indicated in the panel titles (94–97% ANI). The alignment fraction cut-offs, defined as the required percentage of genome length aligned between genome pairs (20–60%), is indicated by line colour. In each panel, the vertical axis indicates the number of OTUs identified from genomes on the basis of the ANI cut-off, alignment fraction cut-off and the degree of incompleteness and/or amount of contamination present in the 296 genomes. **a**, OTUs were identified for the 296 *Bacteroides* genomes with up to 80% of genes randomly removed. The number of OTUs is inflated when genomes are incomplete and the alignment fraction is >20%. **b**, OTUs were identified for the 296 *Bacteroides* genomes with up to 20% of genes from a different one of the 296 genomes. The number of OTUs is not affected by contamination when genomes are complete. **c**, OTUs were identified for the 296 *Bacteroides* genomes with 50% of genes randomly removed and up to 20% of genes from a different one of the 296 genomes, representing a worst-case scenario. The number of OTUs is inflated by contamination when genomes are 50% complete. Using a lower ANI threshold (for example, 94 or 95% versus 96 or 97%) reduces the negative effect of contamination. On the basis of these experiments, we chose an alignment fraction cut-off of 20% and an ANI cut-off of 95% for identifying OTUs from MAGs and reference genomes in the current study.

To explore whether the HGM dataset represented novel taxa, we clustered the 60,664 MAGs plus 145,917 non-redundant reference genomes into species-level operational taxonomic units (OTUs) on the basis of 95% average nucleotide identity (Fig. 2a, Supplementary Tables 9, 10). Although the species concept for prokaryotes is controversial^24^, our operational definition is commonly used^3,4^ and is considered to be a gold standard^25^. We found that our species-level OTUs were consistent with taxonomic annotations from other databases, and that they were robust to genome incompleteness and contamination (Extended Data Figs. 3a–c, 5).

**Extended Data Fig. 6:**
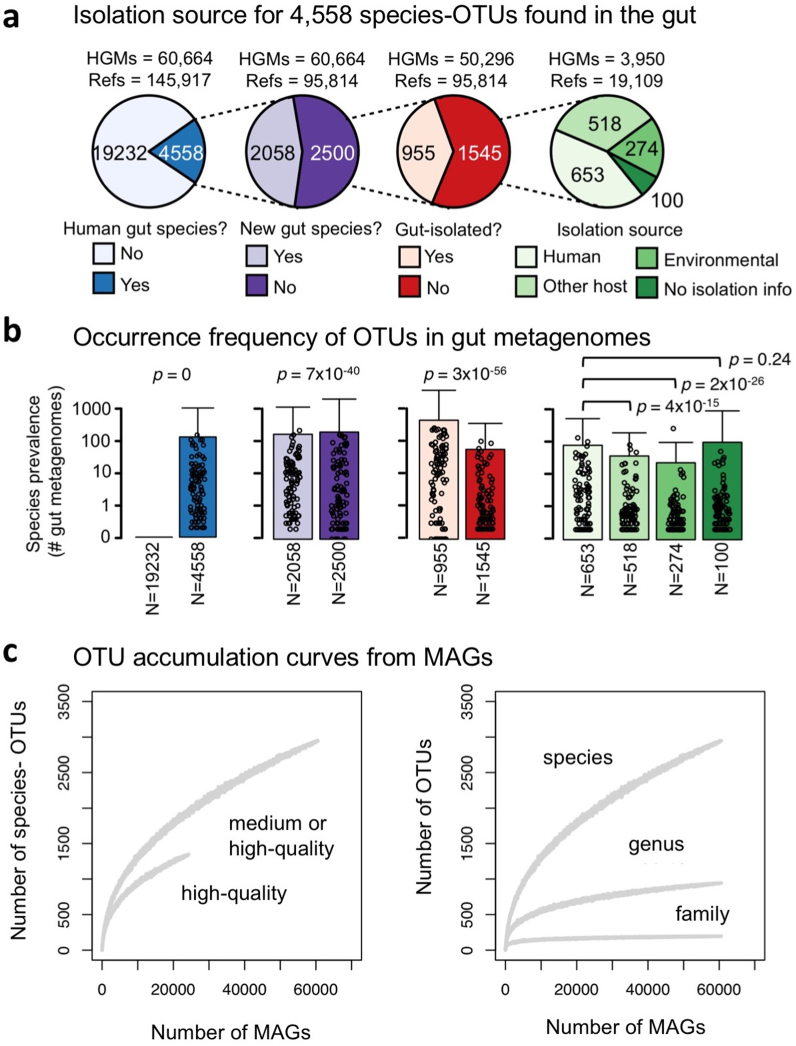
Annotation and accumulation of human gut OTUs. **a**, Of the 23,790 species-level OTUs identified from MAGs and reference genomes, 4,558 were classified as being from the human gut on the basis of (1) having a MAG from the HGM dataset, (2) being detected in a human gut metagenome via read-mapping with IGGsearch or (3) containing a reference genome with metadata that indicate isolation from a human stool sample. Of the 4,558 gut OTUs, 2,058 are represented exclusively by MAGs from the current study and are therefore newly identified. Of the remaining 2,500 represented by reference genomes, only 955 contained a gut-isolated reference genome. The remaining 1,545 OTUs either lack isolation metadata or contain metadata that indicate other isolation sources, including human, non-human and environmental. For example, several gut species from non-host-associated environments were isolated from human food products, including milk, cheese, meat and fermented foods. **b**, The occurrence frequency of all 4,558 gut OTUs was estimated across 3,810 human stool metagenomes using IGGsearch. For bar plots, the centre bar indicates the mean, the error bar indicates the standard deviation and 100 random data points are overlaid. *P* values are from two-sided Wilcoxon rank-sum tests. **c**, Accumulation curves that indicate that the discovery of genus- and family-level OTUs from MAGs has saturated, but that the discovery of species-level OTUs has not. To make the plots, MAGs were randomly sampled without replacement, and the number of unique OTUs was counted for each sample.

Our procedure yielded a total of 23,790 species-level OTUs, which included 4,558 OTUs from the human gut microbiome (Fig. 2a, c, Extended Data Fig. 6a, b, Supplementary Table 10). We formed the Integrated Gut Genomes Database, which contains the 156,478 genomes that comprise the human gut OTUs and includes 2,058 newly identified OTUs that are comprised exclusively of 10,368 MAGs (Fig. 2e). In support of novelty of the newly identified OTUs, 96% of them were not classified at the species level according to the Genome Taxonomy Database^26^ (Supplementary Table 10), and 69% of them had <90% average nucleotide identity to any OTU that contained a reference genome.

A considerable number of MAGs were not taxonomically classified at or above the genus rank (*n* = 3,215) (Supplementary Table 10). To identify the novel clades represented by these MAGs, we constructed a phylogeny of all MAGs and reference genomes, and clustered them on the basis of rank-specific phylogenetic distance cut-offs (Fig. 2b, Extended Data Fig. 3d, e). This revealed 360 genus-level OTUs, 15 family-level OTUs and 2 order-level OTUs that were previously unknown (Fig. 2e). A collector’s curve revealed saturation of OTUs at or above the genus rank, but not for species (Extended Data Fig. 6c). Together, MAGs from the newly identified OTUs represented 70.9% of the total phylogenetic diversity of sequenced gut bacteria, and a 50% increase compared to reference genomes alone (Fig. 2d).

**Fig. 3:**
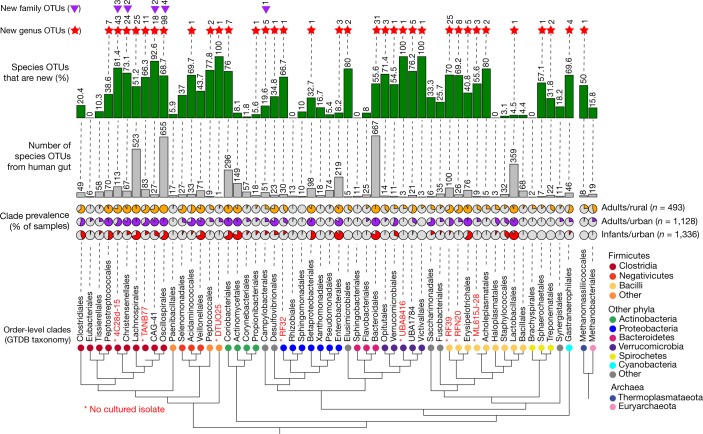
Newly identified gut species are broadly distributed across taxonomic groups. Order-level clades with ≥10 human gut species-level OTUs or that were detected in ≥10% of metagenomes from healthy individuals. Taxonomic labels are based on the Genome Taxonomy Database (GTDB). Red labels indicate orders represented exclusively by MAGs (whether in the current study or from previous studies). Pie charts indicate the prevalence of orders across metagenomes from healthy individuals. Grey bars indicate the number of gut species-level OTUs per order, and the green bars indicate the percentage of OTUs that are newly identified in this study. Red stars and purple triangles indicate the number of newly identified genus-level and family-level OTUs, respectively.

**Extended Data Fig. 7:**
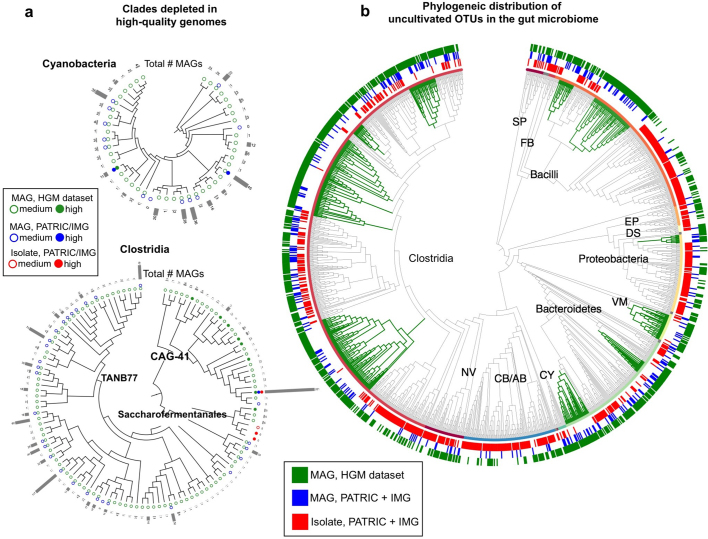
Large lineages are depleted in high-quality genomes and isolate genomes. **a**, The trees indicate the phylogenetic distribution of species-level OTUs from the human gut for Cyanobacteria, and a subclade within Clostridia. All OTUs within the Cyanobacteria phylum were assigned to the Melainabacteria class. Each tip indicates one species-level OTU. Circles indicates whether a medium- (open circle) or high-quality genome (closed circle) was recovered for MAGs from the HGM dataset (green), MAGs from PATRIC + IMG datasets (blue) or isolate genomes from PATRIC + IMG datasets (red). Diversity within these clades would have been missed without the inclusion of medium-quality MAGs. **b**, The tree indicates the phylogenetic distribution of bacterial genus-level OTUs from the human gut (*n* = 1,321 OTUs). The outer rings indicate whether an OTU contains a MAG from the HGM dataset (green), a MAG from PATRIC + IMG dataset (blue) or an isolate genome from PATRIC + IMG dataset (red). Labels indicate phyla. Large monophyletic clades that are depleted in isolate genomes are highlighted with green branches.

The newly identified OTUs were broadly distributed across taxonomic groups (Fig. 3), although with hotspots of diversity in the Firmicutes orders Lachnospirales and Oscillospirales. Nearly 400 OTUs were discovered within the Bacteroidetes, despite the challenges of assembling this phylum (Figs. 1f, 3). By contrast, almost no newly identified OTUs were found in Archaea even though MAGs were easily assembled (Fig. 1f), which suggests that most of the abundant human gut Archaea already have a sequenced genome. Several large clades within Cyanobacteria (Melainabacteria class) and Clostridia were not represented by any high-quality genome, which may be explained by genome reduction or unknown factors that interfere with genome assembly (Extended Data Fig. 7a). Overall, these results indicate that the HGM dataset has greatly expanded the known genomic diversity of bacteria across the tree of life in the human gut.

## Distribution of species in the human population

To quantify the abundance of newly identified OTUs in the gut microbiome, we developed a tool named ‘IGGsearch’, which uses a strategy similar to that of MetaPhlAn2^27^. IGGsearch rapidly estimates the abundance of all 23,790 species-level OTUs, by aligning metagenomic reads to a database of single-copy, species-specific genes that have been identified from MAGs and reference genomes (Supplementary Fig. 1, Methods). A number of other tools exist for metagenomic taxonomic profiling, but none of these contains the MAGs from this study. Using benchmark datasets, we found that IGGsearch accurately quantifies OTU abundance, as well as presence versus absence of OTUs (Supplementary Fig. 2, Supplementary Tables 11, 12).

Using IGGsearch profiling, we found that the newly identified species-level OTUs accounted for 33.4% of richness and 27.7% of relative abundance per sample from healthy individuals (Extended Data Fig. 4c) and were commonly detected in samples from which no MAG was recovered (Extended Data Fig. 4e). These results were consistent in metagenomes that were not used for assembly or binning (Supplementary Table 13). The newly identified species-level OTUs were particularly abundant in healthy adults from rural populations (Tanzania, Peru, Mongolia, Fiji and El Salvador) but were notably rare in infants from Europe and the United States (Fig. 2f, Extended Data Fig. 4d), which may reflect biases in previous genome sequencing efforts. Microbial communities with high diversity were enriched for newly identified OTUs, although no difference was observed between the microbiomes of healthy individuals and individuals with disease (Extended Data Fig. 4f, Supplementary Tables 13, 14). Together, these results reveal that the uncultured OTUs discovered in this study comprise a considerable fraction of the healthy human gut microbiome, and that they are more common in non-Western populations.

## Association of gut species with human diseases

Human gut microbiota have been linked to a myriad of diseases: disease associations with the microbiome can be leveraged to understand disease aetiology, for clinical diagnosis or for building predictive models^1,21^. We hypothesized that IGGsearch would be able to identify associations with human diseases among the 2,058 species-level OTUs discovered in this study. To address this question, we performed metagenome-wide association of 4,558 species-level OTUs from the Integrated Gut Genomes Database versus disease status for ten clinical microbiome studies (including six that were not used for MAG recovery) (Supplementary Tables 15, 16, Methods).

**Fig. 4:**
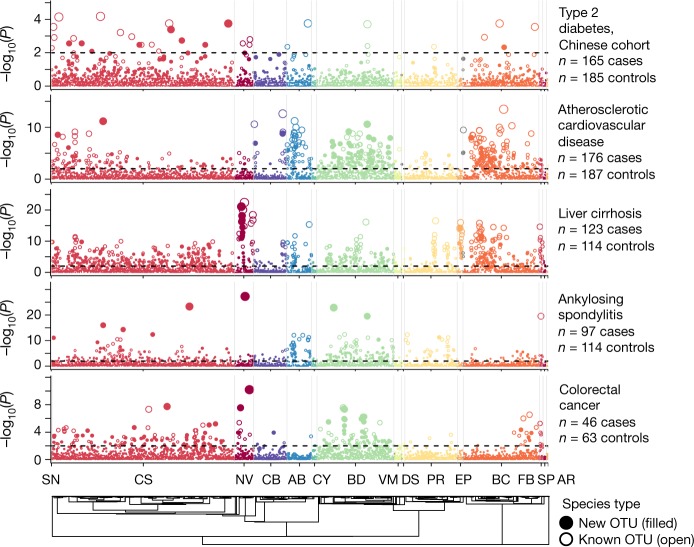
Metagenome-wide association of gut OTUs with human diseases. The Manhattan plot shows the phylogenetic distribution of species–disease associations for different metagenomic studies. Each point is one species-level OTU and point height indicates the *P* value from a two-sided Wilcoxon rank-sum test of estimated species abundance between diseased and healthy individuals after correction for multiple hypothesis tests. The dotted line indicates a false discovery rate of 1%. The plot shows results for five diseases with more than ten species–disease associations. Species are ordered according to their phylogeny, which is displayed at the bottom. AR, Archaea; AB, Actinobacteria; BC, Bacilli; BD, Bacteroidetes; CB, Coriobacteriia; CS, Clostridia; CY, Cyanobacteria; DS, Desulfobacteraeota; EP, Epsilonbacteraeota; FB, Fusobacteria; NV, Negativicutes; PR, Proteobacteria; SN, Synergistetes; SP, Spirochaetes; VM, Verrucomicrobia.

Overall, we identified 2,283 associations between species and disease (at a false discovery rate of <1%) that included an even balance of case-enriched and control-enriched OTUs (Extended Data Table 1). Nearly 40% of the disease associations corresponded to the newly identified OTUs, including many of the most significant associations (Fig. 4). For example, the most significant association for ankylosing spondylitis (an inflammatory arthritis that affects the spine and large joints) was with a newly identified species in the Negativicutes class (OTU 14148, adjusted *P* = 5.3 × 10^−28^), which was strongly depleted in patients relative to healthy controls and eight orders of magnitude more significant than any previously known species.

To contextualize these results, we estimated microbial species abundances in the same datasets using three other commonly used tools—MIDAS^4^, mOTU^3^ and MetaPhlAn2^27^—along with the reference databases that are distributed with each tool. After applying the same statistical procedure to each set of abundance profiles, we identified 716, 404 and 326 disease associations using each respective tool (Extended Data Table 1), which is nearly fivefold fewer than we identified using IGGsearch, on average. Additionally, we used abundance data from each tool to build random-forest machine learning models to predict disease status. We found that IGGsearch abundance profiles yielded the most-predictive model (or equivalent) for eight of the ten diseases, with considerable improvements for colorectal cancer, cardiovascular disease, type 2 diabetes and rheumatoid arthritis (Extended Data Table 1). More work is needed to understand how associated species relate to disease aetiology, and whether these results can be replicated in other human populations.

## Genome reduction of uncultured gut bacteria

Previous MAG studies of environmental communities have uncovered large uncultured lineages with unusual genomic properties, including reduced genomes, slow replication rates and the absence of conserved genes^19,28^. We found that the human gut also contains a number of large lineages that are exclusively represented by MAGs (Fig. 2e, Extended Data Fig. 7b). To elucidate biological properties of these organisms, we performed a comparative genomic analysis between cultured and uncultured species-level OTUs from the gut (Supplementary Table 17, Methods).

**Fig. 5:**
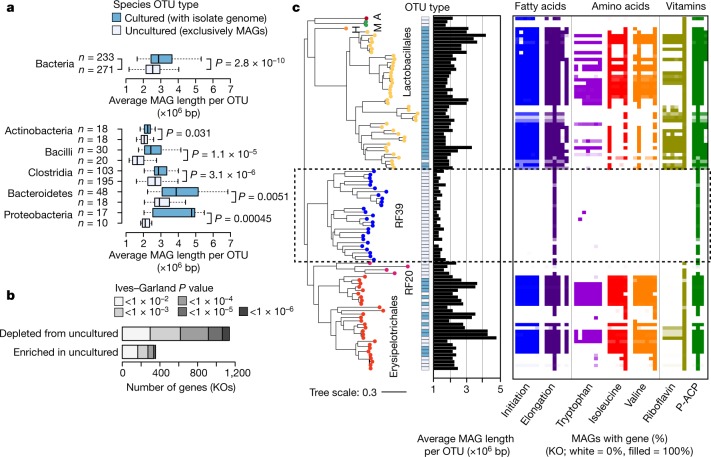
Uncultured OTUs have reduced genomes and are missing common biological functions. **a**, Comparison of genome size between cultivated and uncultivated species-level OTUs after correction for incompleteness and contamination. The middle line of the box plots denotes the median; the box denotes the IQR; and the whiskers denote 1.5× IQR. **b**, Genes from the KEGG database were compared between 233 cultivated and 271 uncultivated species-level OTUs using phylogenetic logistic regression. Most genes associated with cultivated status are depleted from uncultured OTUs. KO, KEGG orthology group. **c**, Phylogenetic tree of species OTUs from Bacilli that were detected in >1% of gut metagenomes. Tip labels and colours indicate order-level clades from the GTDB. A, Acholeplasmatales; M, ML615J-28; H, Haloplasmatales. RF39 has a highly reduced genome with numerous metabolic auxotrophies. P-ACP, pimeloyl-acyl-carrier protein.

**Extended Data Fig. 8:**
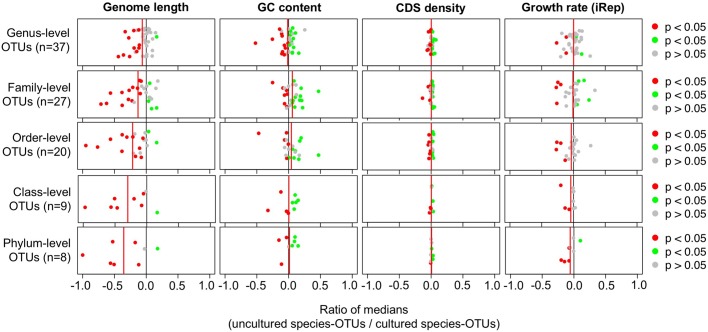
Genome size consistently differs between MAGs from cultivated and uncultivated species-level OTUs, but other features do not. Each column indicates one genomic feature (genome size, GC content, coding density and growth rate) that was compared between high-quality MAGs (*n* = 24,345) from cultivated species-level OTUs (*n* = 233) and MAGs from uncultivated species-level OTUs (*n* = 271). To reduce redundancy, genomic features were averaged across all MAGs per species-level OTU. The value of each point in the figure indicates the log_2_ ratio of each genomic feature between uncultivated species-level OTUs and cultivated species-level OTUs. Each point indicates a single OTU at a higher taxonomic rank, with the rank indicated by row labels (only including higher-rank OTUs with at least ten cultivated and ten uncultivated species-level OTUs). Red and green points indicate whether the distribution of a genomic feature was significantly different between groups based on a two-sided Wilcoxon rank-sum test after correction for multiple hypothesis tests (*α* = 0.05). For example, a value of −1.0 at the phylum level for genome size indicates that the genome size of MAGs within uncultivated species was 2× smaller than for cultivated species within a single phylum. Overall, MAGs from uncultivated species had consistently smaller genomes across taxonomic groups regardless of the taxonomic rank, whereas other genomic features (GC content, coding density and growth rate) did not consistently or systematically differ.

Notably, uncultured OTUs tended to have significantly reduced genomes, a finding that was consistent across all of the major phyla and classes that we tested—including Actinobacteria, Bacilli, Clostridia, Bacteroidetes and Proteobacteria (Fig. 5a). Previous studies have identified difficult-to-culture taxa with reduced genomes—including TM7^29^ and Melainabacteria^30^—but to our knowledge, this is the first time genome reduction has been identified as a broadly shared feature of uncultivated bacteria from the gut microbiome. Other genomic features—including estimated replication rates, coding density and GC content—did not consistently differ between cultured and uncultured OTUs (Extended Data Fig. 8, Supplementary Table 18).

**Extended Data Fig. 9:**
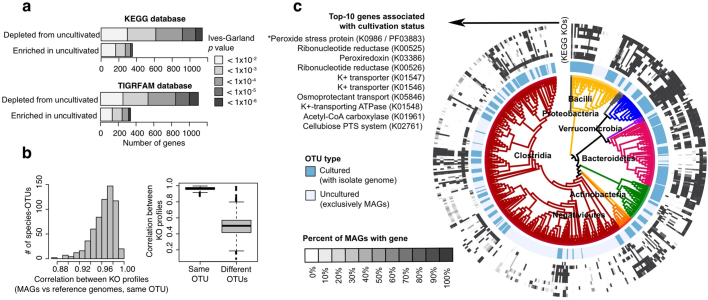
Uncultivated OTUs are depleted in numerous functions, including genes for osmotic and oxidative stress. Genes from high-quality MAGs were functionally annotated on the basis of the KEGG and TIGRFAM databases, and the presence and absence of functions was averaged across MAGs per OTU. Functions were then compared between uncultivated OTUs (*n* = 271) and cultivated OTUs (*n* = 233) using the Ives–Garland phylogenetic logistic regression test, and *P* values were corrected for multiple hypothesis testing using the Benjamini–Hochberg procedure. **a**, The number of genes associated with cultivation status does not depend on the database used for functional annotation. **b**, KEGG functional annotations were compared between high-quality MAGs and reference genomes from the same species-level OTU (left) (*n* = 665 OTUs), and between MAGs and reference genomes from different OTUs using Pearson correlation (right) (*n* = 665 OTUs). MAGs and reference genomes have concordant functional annotations. In the box plots, the middle line denotes the median, the box denotes the IQR and the whiskers denote 1.5× IQR. **c**, Phylogenetic tree of 271 uncultivated OTUs and 233 cultivated OTUs. The inner ring indicates whether an OTU is cultivated or not. The outer ring indicates the presence or absence genes from the KEGG database. The top ten genes associated with cultivation status are shown. Of these, four are related to the maintenance of osmotic pressure (KEGG identifiers K05846, K01547, K01546 and K01548) and two (including the top hit) are related to oxidative stress (identifiers K0986 and K03386). Note that identifier K0986 (the top hit) is listed as encoding an uncharacterized protein in the KEGG database, but as encoding a peroxide stress protein in the PFam database (identifier PF03883). Organisms that lack these functions may have decreased viability during cultivation owing to oxygen exposure and osmotic stress from growth in culture medium.

Given the overall pattern of genome reduction, we identified functions that were frequently missing from uncultured OTUs using phylogenetic logistic regression and annotations from the Kyoto Encyclopedia of Genes and Genomes (KEGG) database (Methods). Overall, we found 1,492 KEGG orthology groups (21.5% of total) that significantly differed between groups at a false discovery rate of <1%—most of which were depleted from uncultivated OTUs (Fig. 5b). These patterns were consistent between MAGs and isolate genomes of the same species, and were not affected by the database used for functional annotation (Extended Data Fig. 9a, b). Among our top hits, we found functions related to the maintenance of osmotic pressure and protection against oxidative stress (Extended Data Fig. 9c), which may indicate that uncultivated bacteria are less viable after transfer to a culture medium or are more sensitive to oxygen exposure outside of the host^6^.

The above patterns were best exemplified by RF39, which is an uncultivated order within the class Bacilli that has a highly reduced genome and numerous auxotrophies (Fig. 5c). Little has been published regarding this group, despite the fact that RF39 has previously been detected in MAG studies^11,20^ and that it was found in a large proportion of metagenomes analysed in our study (Fig. 3). Numerous highly conserved metabolic pathways—including those for biosynthesis of fatty acids, several amino acids and vitamins—were entirely missing from nearly all RF39 genomes. The complete loss of the fatty acid biosynthesis pathway was notable, because fatty acids are integral components of cellular membranes and are considered to be a housekeeping capacity of cells. These organisms may incorporate exogenous fatty acids into membrane phospholipids using a mechanism that has recently been described in Firmicutes^31^.

## Discussion

Here we have illustrated that it is possible to use large-scale metagenomic assembly and binning to recover thousands of genomes for previously unknown members of the human gut microbiome. We generated the Integrated Gut Genomes Database and the IGGsearch tool as resources to drive further discoveries in human microbiome science. During the review of this manuscript, several studies were published that generated many new human gut genomes from metagenomes^32,33^ and cultivated isolates^34,35^. In the future, these genomes could be integrated with the Integrated Gut Genomes Database to provide an updated catalogue of genomes from the gut microbiome.

Although we recovered thousands of MAGs, we also identified several challenges—including low species abundance, high strain diversity and low recovery rates for some phyla (such as Bacteroidetes). Future efforts to recover MAGs from the gut microbiome may benefit from alternative approaches that target these hard-to-assemble organisms. Likewise, we found that adults from non-Western countries were a major source of previously unknown diversity, which indicates that future metagenome studies should focus on human populations outside of Europe, the United States and China.

One of the most surprising results from our study was that the majority of microbial diversity in the human gut is not currently represented by cultured isolates, which are important for numerous applications in basic research and biotechnology. In the future, MAGs from this study could be used to improve culture conditions or identify novel growth factors for uncultured human gut species. For example, menaquinone and fatty acids have been shown to promote the growth of uncultured bacteria^36,37^ and both pathways were missing from many uncultured OTUs from this study (Supplementary Table 19). Furthermore, we found that uncultivated bacteria have undergone considerable genome reduction, which may be an adaptive process that results from use of public goods (as outlined in the Black Queen hypothesis^38^); more work is needed to explore this question.

## Methods

### Publicly available human gut metagenomes

We downloaded 11,523 sequencing runs for publicly available human gut metagenomes from the NCBI SRA^39^. These data correspond to 3,810 samples, 15 studies^9,21,40–51^ (and https://olive.broadinstitute.org/projects/infant_gut_flora_and_antibiotics) and >181 billion sequencing reads with an average length of 100 bp (Supplementary Tables 1, 2). Sequencing metadata were obtained from the SRAdb relational database^52^ and host metadata were obtained from either the NCBI BioSample database^53^ or from supplementary datasets linked to publications (Supplementary Table 3). No metadata were available online or upon request from the Fiji cohort^9^; these individuals were treated as healthy adults from a rural population.

### Metagenome assembly and binning

We co-assembled the 11,523 sequencing runs for each of the 3,810 biological samples using MegaHIT v.1.1.1^54^ with default parameters. This resulted in 333,661,332 contigs longer than 200 bp, totalling 453.5 × 10^9^ bp, with an average N50 of 12,460 bp (Supplementary Table 2). Human gut MAGs were generated per sample using three different tools with default options: MaxBin v.2.2.4^55^, MetaBAT v.2.12.1^56^ and CONCOCT v.0.4.0^10^, which all use a combination of sequence composition and coverage information. DAS Tool v.1.1.0^57^ with option ‘-score_threshold 0’ was used to integrate MAGs produced by the three tools. Contigs shorter than 1 kb were discarded. This process resulted in 152,591 MAGs longer than 100 kb, which totalled 73,632,219 contigs (22% of total assembled) and 310.7 × 10^9^ bp (69% of total assembled). All MAGs were screened for contamination against the human genome (build 38) and phiX genome with BLASTN v.2.6.0^58^.

### Refinement of MAGs on the basis of alignment of contigs between conspecific genomes

To refine MAGs from the HGM dataset, we performed pairwise alignment of contigs between MAGs and other closely related, near-complete MAGs and reference genomes (Supplementary Table 6). Our logic was that strains of the same species should share homology between most contigs, and that contigs that fail this condition (that is, are present in one genome but absent in the other) probably represent contamination. For each input MAG, we used Mash v.2.0^59^ to find at least five closely related, near-complete genomes in the database (>95% estimated completeness, <5% estimated contamination, Mash distance ≤ 0.05, *P* ≤ 0.001), and then used BLASTN to align contigs between each MAG and all target genomes. Contigs in the MAG that failed to align at ≥70% nucleotide identity over ≥25% length to any of the closely related genomes were flagged for removal.

### Refinement of MAGs on the basis of taxonomic annotation of contigs

We identified and removed taxonomically discordant contigs from MAGs using two complementary approaches (Supplementary Table 6). The first approach performs taxonomic annotation on the basis of universal single-copy marker genes. Hidden Markov models for marker-gene families were downloaded from the PhyEco database^60^, and searched against MAGs with HMMER v.3.1b2^61^. A subset of 100 (for Archaea) or 88 (for Bacteria) gene families was used. Marker genes found in MAGs were then aligned against a reference database of taxonomically annotated marker genes from reference genomes using BLASTP. For each gene, we transferred the taxonomy of the best hit in the reference database at the appropriate rank on the basis of the percentage of amino acid identity cut-offs specific to each gene family at each rank. We then taxonomically annotated each MAG on the basis of the consensus taxonomy of marker genes at the lowest rank, such that >70% of marker genes were annotated. Contigs were flagged for removal if they (1) contained a taxonomically discordant marker gene, and (2) lacked a concordant marker gene. The second approach for taxonomic refinement is similar to the first, except that 855,764 clade-specific prokaryotic marker genes from the MetaPhlAn2^27^ database were used for taxonomic annotation after excluding ‘pseudo markers’ that are not unique to a clade.

### Refinement of MAGs on the basis of outlier nucleotide composition and sequencing read depth

Using an approach similar to a previously published method^11^, we identified and removed contigs from MAGs with either (1) outlier GC content, (2) outlier tetranucleotide frequency or (3) outlier sequencing read depth (Supplementary Table 6). We used principal component analysis to reduce the tetranucleotide frequency dimensionality down to the first principal component (tetranucleotide frequency PC1). For each MAG, we then measured the average GC content, average tetranucleotide frequency PC1 and average sequencing read depth. Contigs were flagged for removal if they deviated from these averages beyond cut-offs selected to minimize reduction in completeness (Supplementary Table 6).

### Validation of MAG refinement pipeline

We simulated 1,000 human gut MAGs to validate our overall MAG refinement strategy (Supplementary Table 7). Each simulated MAG contained two genomes: one ‘host’ genome (representing the target genome) and one ‘donor’ genome (representing the contaminating genome). All 102 genomes used in simulations were isolated from the human gut, and were estimated to have >95% completeness, <1% contamination and <25 contigs. MAGs were simulated with completeness (mean = 61.9%), contamination (mean = 10.0%) and N50 (mean = 35.8 kb) on the basis of randomly sampled MAGs from the HGM dataset. MAGs were dropped in cases in which contamination exceeded completeness, and thus the host genome was in the minority. The refinement pipeline was applied to each simulated MAG and—to evaluate the pipeline—we quantified the overall reduction in completeness and contamination (Extended Data Fig. 1a, b).

### Application of refinement strategies to the HGM dataset

We applied each of the refinement approaches described above to the MAGs (Extended Data Fig. 2c, Supplementary Table 6). In rare cases, these approaches may erroneously flag a large proportion of a MAG. To avoid this, we applied a particular approach to a MAG only if it resulted in ≤25% reduction in total length. The five approaches combined removed 5,251,859 contigs (7.13% of total) and 20,821.2 Mb (6.70% of total) from the MAGs. After removing potential contaminants, we were left with 152,279 MAGs with a total length ≥100 kb and 10,036 individual contigs longer than 100 kb that were either unbinned or removed during decontamination. These long contigs were included with other MAGs, which brought the total number to 162,315.

### Quality assessment of MAGs

CheckM v.1.0.7^13^ was used to estimate completeness and contamination of the 162,315 recovered MAGs (Supplementary Table 6); CheckM is based on the copy-number of lineage-specific single-copy genes. Additional statistics were obtained for each genome, including the contig N50, number of contigs, average contig length, contig read-depth, and number of tRNA and rRNA genes. tRNAs were identified using tRNAscan-s.e. v.1.3.1^62^ and rRNA genes using Barrnap v.0.9-dev^63^ with options ‘–reject 0.01 –evalue 1e-3’. We identified 60,664 MAGs that met the MIMAG medium-quality criteria of ≥50% completeness with ≤10% contamination^14^. For analyses that required near-complete genomes, we used a subset of 24,345 high-quality MAGs that were ≥90% complete, ≤5% contaminated, with an N50 ≥ 10 kb, an average contig length ≥5 kb, ≤500 contigs and ≥90% of contigs with ≥5× read-depth.

### Estimation of SNP density

Read mapping and SNP calling were performed to assess the genetic diversity of each MAG (Supplementary Table 5). Bowtie 2 v.2.3.4^64^ was used to construct a database of MAGs for each sample, and to align metagenomic reads. Reads with low mapping and sequence quality were discarded (quality scores <20 and <30, respectively), and we counted the occurrence of nucleotides with quality ≥30 across each MAG. To compare SNPs between MAGs sequenced to different depths, we down-sampled each MAG to 40 mapped reads per site. MAGs with at least 200,000 sites of ≥40× depth were retained for analysis. A SNP was called if at least two reads matched the alternative allele at a genomic site. SNP density was calculated as the number of SNPs per kilobase.

### Reference genomes used for comparison

We downloaded 201,102 publicly available bacterial and archaeal reference genomes from the Integrated Microbial Genomes (IMG; https://img.jgi.doe.gov/)^65^ (*n* = 61,713) and Pathosystems Resource Integration Center (PATRIC; https://www.patricbrc.org/)^66^ (*n* = 139,389) databases, on 16 January 2018. These included genomes from 2 human gut culturomics studies^6,7^ and 16,525 previously published MAGs, including a previous MAG study from the human gut^20^ and nearly 8,000 MAGs assembled from SRA metagenomes^11^. To remove redundancy within and between databases, we used Mash^59^ with default parameters to cluster genomes with a Mash distance of 0.0, which are expected to be identical. This resulted in 153,900 non-redundant reference genomes, of which 127,419 were classified as high quality, 18,498 as medium quality and another 7,983 as low quality (Supplementary Table 9).

### Species-level clustering of reference genomes and MAGs

Using an approach similar to a previously published method^67^, we clustered the 60,664 MAGs and 145,917 reference genomes meeting or exceeding the MIMAG medium-quality standard into species-level OTUs on the basis of 95% whole-genome ANI (Supplementary Table 10). We first performed single-linkage clustering of genomes on the basis of a Mash ANI of 99%, which resulted in 79,675 clusters that can be confidently assigned to the same species-level OTU. Mash is extremely fast, although it can underestimate ANI for incomplete genomes^67^. To address this, we used the ANIcalculator v.1.0^68^ to compute whole-genome-based ANI (gANI) between the 99%-identity clusters, and required that at least 20% of genes were aligned. The 20% cut-off was chosen to minimize the negative effects of incomplete genomes, and to avoid the formation of spurious OTUs (Extended Data Fig. 5a). To increase computational efficiency, we calculated gANI only for genome pairs with >90% Mash ANI. Genomes were clustered into OTUs using average-linkage hierarchical clustering with a 95% gANI cut-off using the package MC-UPGMA v.1.0.0^69^, which yielded 23,790 OTUs.

All OTUs were taxonomically annotated using the tool GTDBTk v.0.0.6 (release 80, www.github.com/Ecogenomics/GtdbTk), which produces standardized taxonomic labels that are based on those used in the Genome Taxonomy Database^26^. Additionally, we constructed pan-genomes on the basis of clustering all genes within each OTU, using VSEARCH v.2.4.3^70^ with 90% DNA identity and 50% alignment cut-offs (maximum 500 genomes per OTU). Human gut OTUs were identified from the set of 23,790 OTUs on the basis of (1) containing a MAG from the HGM dataset, (2) being detected by IGGsearch (see ‘Development of IGGsearch for metagenomic profiling of species-level OTUs’) in at least 1 of 3,810 metagenomes used for MAG recovery or (3) containing a genome isolated from the human gut (Extended Data Fig. 6a, b, Supplementary Table 10). A total of 4,558 species-level OTUs were annotated as being found in the human gut, on the basis of a combination of the three criteria.

### Phylogenetic analysis of MAGs and reference genomes

We constructed phylogenetic trees of MAGs and reference genomes using concatenated alignments of conserved, single-copy marker-gene families from the PhyEco database^60^ for Bacteria (*n* = 88 genes) and Archaea (*n* = 100 genes). Individual marker genes were identified using HMMER v.3.1b2 with bit-score cut-offs that are specific to gene family. For computational efficiency, genomes were collapsed down to species-level OTUs, which were represented as individual leaves in the phylogenetic tree. To reduce the effect of contamination, taxonomically discordant marker genes were removed, as described in ‘Refinement of MAGs on the basis of taxonomic annotation of contigs’. FAMSA v.1.2.5^71^ was used to construct protein-based multiple sequence alignments for each gene family. Columns with >15% gaps were removed, alignments were concatenated and sequences with >70% gaps were removed (*n* = 39). FastTree2 v.2.1.10^72^ was used to build a maximum likelihood phylogeny for Bacteria and Archaea with default options. All trees were visualized using iTOL v.3^73^. To quantify the gain in phylogenetic diversity from the HGM dataset, we computed the total branch length of two subtrees: a tree of all 4,558 gut OTUs (PD_Gut_) and a tree of 2,500 gut OTUs with reference genomes (PD_RefGut_). The percentage gain in phylogenetic diversity was computed as: 100 × (PD_Gut_ − PD_RefGut_)/PD_RefGut_. To identify OTUs for higher-rank groups, we performed average-linkage hierarchical clustering of phylogenetic distances, which was implemented in R (Supplementary Table 10). Rank-specific cut-offs were identified by maximizing similarity to the Genome Taxonomy Database for reference genomes (Extended Data Fig. 3d, e).

### Development of IGGsearch for metagenomic profiling of species-level OTUs

Using an approach similar to MetaPhlAn2^27^, we developed an accurate and efficient tool for quantifying the abundance of species-level OTUs from unassembled metagenomes. First, we identified marker genes for each OTU (Supplementary Fig. 1a). Up to 300 genes from the pan-genome of each OTU were selected with the maximum intra-OTU frequency and minimum inter-OTU frequency. The intra-OTU frequency was computed as the fraction of genomes within an OTU in which a gene was found at 90% DNA identity. The inter-OTU frequency was determined on the basis of DNA alignments (using HS-BLASTN v.0.0.5^74^) between each gene and the pan-genomes of other OTUs, and accounts for (1) the number of other pan-genomes in which the gene is found, (2) the frequency of the gene in each pan-genome and (3) the percentage of identity of each alignment. For computational reasons, genes were first aligned within each phylum, and only the 300 top-scoring candidates per OTU were subsequently checked for uniqueness between phyla. A total of 6,198,663 marker genes were identified for 23,790 OTUs.

A large number of OTUs contained just a single genome, which made it difficult to accurately predict conserved genes. To refine our marker-gene set, we used abundance co-variation information, which is a common strategy for binning genetic regions from the same species and has previously been applied^3,10,20,21,56^. Specifically, we performed read-mapping of the 3,810 metagenomic samples to the database of 6,198,663 marker genes using Bowtie 2 v.2.3.4 and quantified the read depth of each gene in each sample. We used average linkage clustering to group genes from each OTU into co-variance groups on the basis of Pearson correlations of read depth across samples (Supplementary Fig. 1b). After applying a correlation threshold of 0.90, we selected the largest cluster of genes for the final marker-gene set. This procedure removed 55,132 genes for 1,402 OTUs that were present in ≥10 samples with ≥1× coverage.

IGGsearch is a command-line tool that uses Bowtie 2 to map metagenomic reads to the database of marker genes and quantify species-level OTUs. Read alignments are removed with low percentage of identity (minimum = 95%), alignment coverage (minimum = 70% of read) and base quality (minimum = 20). For each metagenomic sample, OTU relative abundance is estimated by taking the average read depth across marker genes and normalizing these values to 1.0 across all OTUs. Species presence is determined on the basis of the percentage of marker genes with at least one mapped read.

The sensitivity and specificity of IGGsearch was evaluated on two benchmark datasets. First, we benchmarked IGGsearch on the CAMI challenge dataset (https://data.cami-challenge.org/participate) (Supplementary Tables 11, 12, Supplementary Fig. 2a). Second, we benchmarked IGGsearch on simulated gut metagenomes that contained between 500,000 and 50,000,000 paired-end reads, read length of 100 bp, Illumina-style sequencing error and 1 genome from each of 100 randomly selected gut species-level OTUs (Supplementary Fig. 2b). On the basis of these benchmarks, we called OTUs present when at least 15% of their marker genes were detected, which gave a good balance between sensitivity and specificity.

### Metagenome-wide association of species abundance with disease

We used IGGsearch species profiles to identify species-level OTUs associated with disease for ten previously published studies, including colorectal cancer^43^, type 2 diabetes^21,44^, rheumatoid arthritis^42^, Parkinson's disease^75^, atherosclerotic cardiovascular disease^76^, ankylosing spondylitis^77^, non-alcoholic fatty liver disease^78^, liver cirrhosis^79^ and obesity^80^ (Extended Data Table 1, Supplementary Tables 15, 16). To identify species–disease associations, we compared the relative abundances of species for the 4,558 human gut species-levels OTUs between cases and healthy controls using the Wilcoxon rank-sum test. Non-gut OTUs were excluded to reduce the effect of multiple hypothesis testing. For each disease, *P* values were corrected for multiple hypothesis tests using the Benjamini–Hochberg procedure. We performed the same statistical procedure using species profiles from three other tools: MIDAS v.1.3.0^4^, MetaPhAn2 v.2.7.7^27^ and mOTU v.1.1.1^3^. All tools were run with default parameters and the distributed reference data. To prevent confounding signals owing to disease treatment, we excluded 100 individuals taking drugs that affect microbiome composition, including metformin in patients with type 2 diabetes^21,44^, acarbose, atorvastatin, fondaparinux and metoprolol in patients with atherosclerotic cardiovascular disease^76^, and antirheumatic drugs in patients with rheumatoid arthritis^42^.

### Machine learning models for disease prediction

We constructed random forest models implemented in the scikit-learn Python package (https://scikit-learn.org) to predict disease state from species abundance profiles generated with IGGsearch, MIDAS, mOTU and MetaPhlAn2 (Extended Data Table 1). For IGGsearch, we included all 23,790 species OTUs and allowed the random forest model to choose the most-predictive OTUs. Random forest models were implemented in the scikit-learn package v.0.19.1^81^ and were optimized for each of the four tools for each of the ten diseases. Specifically, we tested 1,000 random combinations of parameter values for (1) the number of trees in the forest, (2) the number of features to consider at each split, (3) the maximum number of levels in each tree, (4) the minimum number of samples to split a node, (5) the minimum number of samples at each leaf and (6) whether to use bootstrapping during model training. To avoid overfitting, each model was evaluated using tenfold cross-validation and the combination of parameters that yielded the best receiver operating curve (ROC) area under the curve (AUC) was selected. To obtain robust estimates of model performance, all models were re-run 100 times and ROC AUC values were averaged across runs.

### Identifying genomic features and auxotrophies of uncultured gut bacteria

We selected a subset of 504 human gut species-level OTUs from bacteria for comparative genomic analysis between cultured and uncultured organisms (Supplementary Table 17). OTUs with <5% prevalence in human gut metagenomes were excluded, because rare organisms may be amenable to cultivation but not yet sampled. Uncultivated OTUs were defined as those that contain only MAGs (either from the current study or previous studies, *n* = 271) and cultivated OTUs as those that contain at least one isolate genome (*n* = 233). We based all comparative analysis between OTUs using 24,345 high-quality MAGs from the HGM dataset, which was done (1) to avoid biases that result from a comparison of MAGs to isolate genomes (which differ in assembly quality) and (2) to avoid issues arising from low completeness among MAGs in the medium-quality tier.

We compared several broad genomic features between groups, including estimated genome size, GC content, coding density and estimated replication rate. Estimated genome size was corrected for completeness and contamination using: *Ĝ* = *G* × 100/*Ĉ* − (*G* × $\hat{T}$/100), in which *Ĝ* is the estimated genome size of a MAG, *G* is the observed genome size, *Ĉ* is the estimated percentage completeness and $\hat{T}$ is the estimated percentage contamination. Replication rate was estimated with iRep v.1.10^28^ for MAGs with >5× read-depth, which is based on differences in sequencing depth between the origin and terminus of replication. Genomic features were averaged across all high-quality MAGs for each OTU, and then compared between OTUs using the Wilcoxon rank-sum test (Supplementary Table 18).

To identify potential auxotrophies, we compared the prevalence of genes, modules and pathways from the KEGG database (release 77.1)^82^ between cultivated and uncultivated OTUs. Proteins from high-quality MAGs were annotated on the basis of amino acid alignments to KEGG using LAST v.828^83^, and assigned to the KEGG orthology group with lowest value of *E* < 1 × 10^−5^. Next, we computed the fraction of MAGs per OTU that contained each KEGG orthology group, and compared these values between OTUs using the Ives–Garland test implemented in the phylolm R package v.2.6^84^. The Ives–Garland test performs logistic regression while controlling for differences in phylogeny between groups, and has previously been applied to microbiome data^85^. This analysis was repeated for modules and pathways from the KEGG database. *P* values were corrected for multiple hypothesis tests using the Benjamini–Hochberg procedure (Supplementary Table 19). This same analysis was performed for functions from the TIGRFAM database (release 15.0)^86^ (Extended Data Fig. 9a).

### Reporting summary

Further information on research design is available in the Nature Research Reporting Summary linked to this paper.

## Online content

Any methods, additional references, Nature Research reporting summaries, source data, statements of data availability and associated accession codes are available at 10.1038/s41586-019-1058-x.

### Supplementary information

Supplementary Figures This file contains Supplementary Figures 1 and 2.

Reporting Summary

This file contains legends for Supplementary Tables 1-20.

Supplementary Tables 1-20 This file contains Supplementary Tables 1-20; see accompanying Supplementary Information file for full legends.

## Figures and Tables

**Extended Data Table 1:**
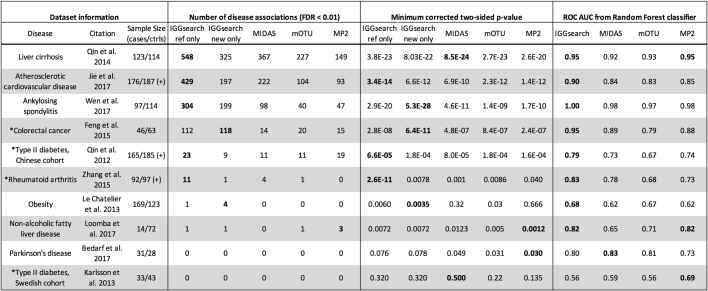
Metagenome-wide disease associations using IGGsearch and other tools

IGGsearch and three other tools—MIDAS, mOTU and MetaPhlAn2 (abbreviated as MP2)—were used to estimate the abundance of species across samples that span ten studies. Two-sided Wilcoxon rank-sum tests were used to identify differentially abundant species and *P* values were corrected for multiple hypothesis testing using the false discovery rate procedure. For IGGsearch, disease associations are split into ‘ref only’ (species-level OTUs with reference genomes) and ‘new only’ (species-level OTUs with only MAGs). Additionally, species profiles from all four tools were used to train random-forest machine learning classifiers to predict disease state. Optimized models were identified by testing 1,000 random forests with random combinations of model parameters, and choosing the model with the greatest ROC AUC. To avoid overfitting, tenfold cross-validation was performed. Reported AUC values are averages across 100 random forest runs. Bold text indicates the tool that performed best for each disease; asterisks indicates studies used for MAG recovery. (+), studies in which a subset of cases was excluded owing to medication for disease treatment.

## Data Availability

Representative MAGs for the 2,058 new species have been deposited in the European Nucleotide Archive (ENA) under accession PRJEB31003 (Supplementary Table 20). The entire HGM dataset, phylogenomic trees and related metadata are available at https://github.com/snayfach/IGGdb.

## Code availability

IGGsearch and the database of conserved species-specific marker genes are available at https://github.com/snayfach/IGGsearch. The code for removing contamination from genome bins (MAGpurify) is available at https://github.com/snayfach/MAGpurify.
