# Supplementary material for: New insights from uncultivated genomes of the global human gut microbiome
Source: Nature. 2019 Mar 13;568(7753):505–10. doi: 10.1038/s41586-019-1058-x (PMC6784871; doi:10.1038/s41586-019-1058-x)
Supplement: Supplementary file 1 — This file contains Supplementary Figures 1 and 2. [file 41586_2019_1058_MOESM1_ESM.pdf]

nature
SUPPLEMENTARY INFORMATION
https://doi.org/10.1038/s41586-019-1058-x
In the format provided by the authors and unedited.

New insights from uncultivated genomes of the global human gut microbiome

Stephen Nayfach[1,2*], Zhou Jason Shi[3,4], Rekha Seshadri[1,2], Katherine S. Pollard[3,4,5,6,7,8] & Nikos C. Kyrpides[1,2*]

[1]United States Department of Energy Joint Genome Institute, Walnut Creek, CA, USA. [2]Environmental Genomics and Systems Biology Division, Lawrence Berkeley National Laboratory, Berkeley, CA, USA. [3]Gladstone Institutes, San Francisco, CA, USA. [4]Chan-Zuckerberg Biohub, San Francisco, CA, USA. [5]Institute for Human Genetics, University of California San Francisco, San Francisco, CA, USA. [6]Institute for Computational Health Sciences, University of California San Francisco, San Francisco, CA, USA. [7]Quantitative Biology Institute, University of California San Francisco, San Francisco, CA, USA. [8]Department of Epidemiology and Biostatistics, University of California San Francisco, San Francisco, CA, USA. *e-mail: snayfach@lbl.gov; nckyrpides@lbl.gov

<u>**Supplementary Figures**</u>

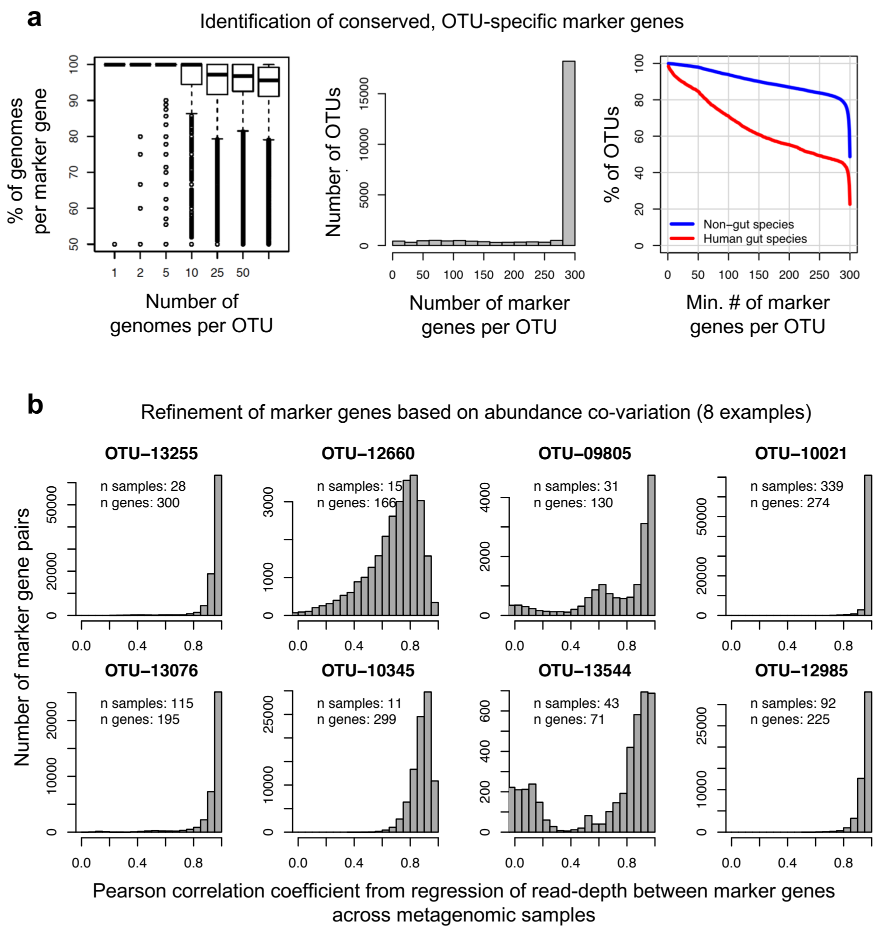

Supplementary Figure 1 | Identification and refinement of conserved OTU-specific marker genes. A) Up to 300 marker genes were identified for each of the 23,790 species-level OTUs, resulting in a total of 6,198,663 marker genes. Over 99% of marker genes are perfectly unique, with 1% found in a small percentage of other species. (Left) The box plot shows the intra-species frequency of the 6,198,663 marker genes. Values close to 100% indicate a marker gene is found in every genome of OTU. In the box plot, middle line denotes median; box denotes IQR; and whiskers denote 1.5× IQR. (Middle) Most OTUs have a full set of 300 marker genes. (Right) Human gut OTUs have fewer marker genes than OTUs from non-gut environments, which may be a result of (1) there are many closely related OTUs from the human gut, making it more challenging to identify unique markers, and (2) non-gut OTUs have fewer genomes per OTU, resulting in more marker genes found across all genomes by chance. B) Example of co-variance-based marker-gene refinement for eight human gut species. All marker genes are expected to be present in a metagenome when an OTU is present and absent when the OTU is absent. Metagenomic read-mapping was performed to identify and remove marker genes failing this condition: 3,810 human gut metagenomes were mapped to the 6,198,663 marker genes for 23,790 OTUs and 1,402 OTUs were detected in >10 samples with >1x depth and were subjected to co-variance-based refinement. The histograms show the distribution of Pearson correlation coefficients of read-depth between marker genes across samples for eight randomly chosen OTUs. The sample size of each histogram is indicated by the number of pairwise comparisons between marker genes (see panel legends).

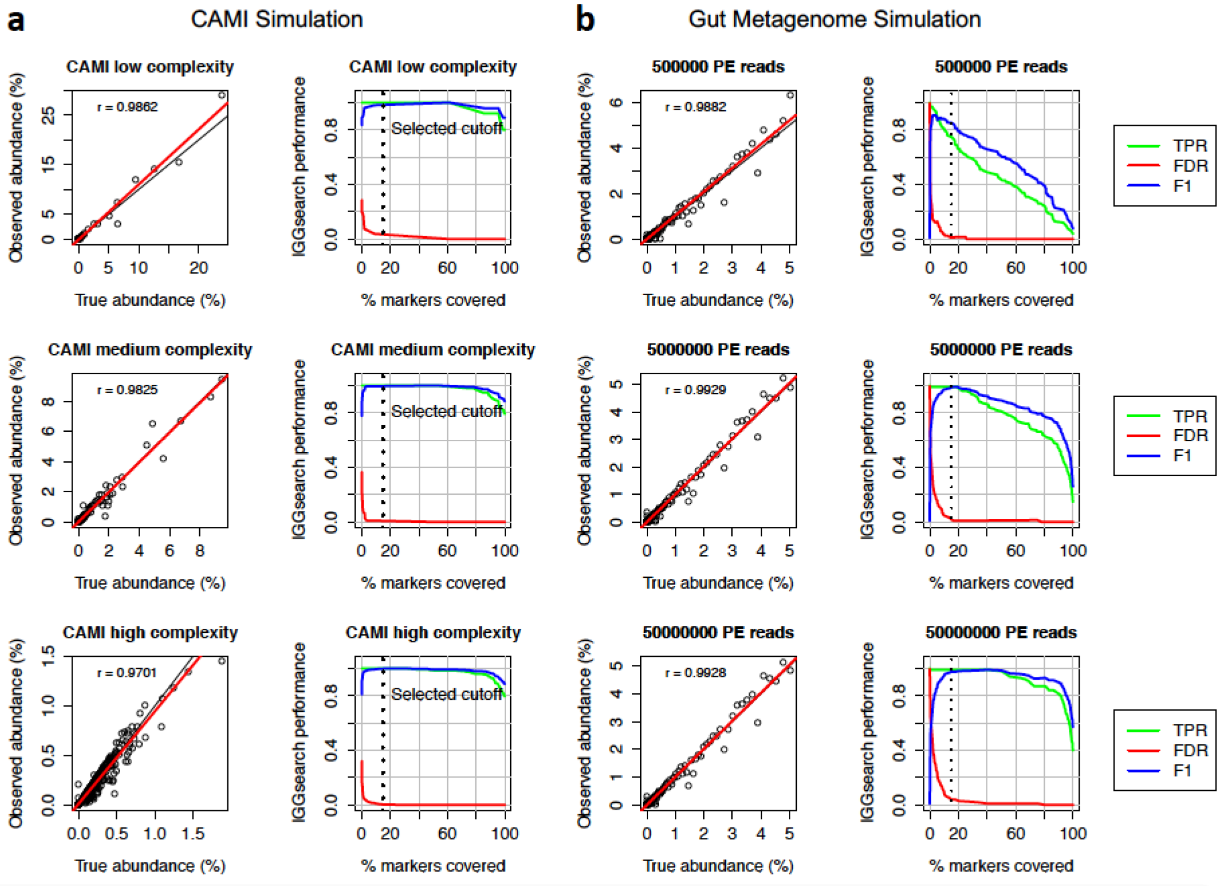

Supplementary Figure 2. IGGsearch accurately estimates OTU presence and abundance in benchmark datasets. A) IGGsearch was applied to metagenomes from the first CAMI challenge (https://data.cami-challenge.org/participate). The low, medium, and high complexity datasets contained 60, 232, and 1,074 genomes respectively. Of these, 35, 128, and 588 could be assigned to one of the OTUs in the IGGsearch database based on a gANI value of >95%. (Left) Scatterplots of true versus estimated relative abundance for all 23,790 OTUs in the IGGsearch database. Red regression lines are from Pearson correlations. (Right) The presence-absence of all 23,790 species OTUs in the IGGsearch database was predicted at different cutoffs, defined at the % of marker genes detected per OTU. The green line indicates the true positive rate (TPR), or the fraction of OTUs present in the metagenome that were detected at a given cutoff. The red line indicates the false discovery rate (FDR), or the fraction of detected OTUs that were not present. The green line indicates the F1-score, or the harmonic mean between the TPR and FPR. The vertical dotted line at 15% indicates the cutoff we selected for prediction of OTU presence, which maximizes F1-score and indicates an optimal balance between the TPR and FDR. B) To evaluate the effect of sequencing depth we constructed mock metagenomes containing between 500,000 and 50,000,000 100-bp paired-end (PE) sequencing reads with an Illumina error model. Each metagenome contained 100 isolate reference genomes from 100 distinct OTUs that were exponentially distributed with a maximum abundance of 5%. (Left and Right) Figures follow the same format as in (A).
