# Supplementary material for: New insights from uncultivated genomes of the global human gut microbiome
Source: Nature. 2019 Mar 13;568(7753):505–10. doi: 10.1038/s41586-019-1058-x (PMC6784871; doi:10.1038/s41586-019-1058-x)
Supplement: Supplementary file 2 — Reporting Summary [file 41586_2019_1058_MOESM2_ESM.pdf]

nature research

Corresponding author(s): Stephen Nayfach, Katherine Pollard, Nikos Kyrpides

Last updated by author(s): Feb 27, 2019

Reporting Summary

Nature Research wishes to improve the reproducibility of the work that we publish. This form provides structure for consistency and transparency in reporting. For further information on Nature Research policies, see Authors & Referees and the Editorial Policy Checklist.

Statistics

For all statistical analyses, confirm that the following items are present in the figure legend, table legend, main text, or Methods section.

n/a	Confirmed	
☐	☒	The exact sample size (*n*) for each experimental group/condition, given as a discrete number and unit of measurement
☐	☒	A statement on whether measurements were taken from distinct samples or whether the same sample was measured repeatedly
☐	☒	The statistical test(s) used AND whether they are one- or two-sided<br>*Only common tests should be described solely by name; describe more complex techniques in the Methods section.*
☐	☒	A description of all covariates tested
☐	☒	A description of any assumptions or corrections, such as tests of normality and adjustment for multiple comparisons
☐	☒	A full description of the statistical parameters including central tendency (e.g. means) or other basic estimates (e.g. regression coefficient) AND variation (e.g. standard deviation) or associated estimates of uncertainty (e.g. confidence intervals)
☐	☒	For null hypothesis testing, the test statistic (e.g. *F*, *t*, *r*) with confidence intervals, effect sizes, degrees of freedom and *P* value noted<br>*Give P values as exact values whenever suitable.*
☒	☐	For Bayesian analysis, information on the choice of priors and Markov chain Monte Carlo settings
☒	☐	For hierarchical and complex designs, identification of the appropriate level for tests and full reporting of outcomes
☐	☒	Estimates of effect sizes (e.g. Cohen's *d*, Pearson's *r*), indicating how they were calculated

Our web collection on statistics for biologists contains articles on many of the points above.

Software and code

Policy information about availability of computer code

Data collection	Assemblies were generated using MegaHIT v1.1.1. MAGs were generated using Maxbin v2.2.4, MetaBAT v2.12.1, CONCOCT 0.4.0, and DAS Tool v1.1.0 (option: '-score_threshold 0'.
Data analysis	MAGpurify v1.0 was used to refine MAGs. CheckM v1.0.7 was used to estimate MAG quality. BLASTN v.2.6.0 was used to remove contigs matching the human genome and phiX genome. HMMER v3.1b2 was used to identify marker genes. tRNAs and rRNAs were identified using tRNAscan-SE v.1.3.1 and Barrnap v0.9-dev (options: '–reject 0.01 –evalue 1e-3'), respectively. Bowtie 2 v2.3.4 was used for aligning reads to each MAG. Mash v2.0, ANIcalculator v1.0, and MC-UPGMA v1.0.0 were used to compute ANI and cluster genomes. GTDBTk v0.0.6 was used to taxonomically annotate MAGs. VSEARCH v2.4.3 (options: '-id 0.9, -target_cov 0.5 -query_cov 0.5) was used to construct pan-genomes. IGGsearch v1.0 was used to estimate the presence-absence and abundance of OTUs from metagenomes. FAMSA v1.2.5 was used for multiple sequence alignment. FastTree2 v.2.1.10 was used for tree building. HS-BLASTN v0.0.5 was used for performing alignment of pan-genome genes between species. IGGsearch was compared to MIDAS v1.3.0, MetaPhAn2 v2.7.7, and mOTU v1.1.1. Machine learning was performed using the scikit-learn package v0.19.1. iRep v1.10 was used to estimate replication rate. LAST v828 was used for alignment against the KEGG database. The phylolm R package v.2.6 was used for phylogenetic logistic regression.

For manuscripts utilizing custom algorithms or software that are central to the research but not yet described in published literature, software must be made available to editors/reviewers. We strongly encourage code deposition in a community repository (e.g. GitHub). See the Nature Research guidelines for submitting code & software for further information.

Data

Policy information about availability of data

All manuscripts must include a data availability statement. This statement should provide the following information, where applicable:

- Accession codes, unique identifiers, or web links for publicly available datasets
- A list of figures that have associated raw data
- A description of any restrictions on data availability

Representative MAGs for the 2,058 new species have been deposited in the European Nucleotide Archive (ENA) under accession PRJEB31003. The entire data set and related metadata is freely available at https://github.com/snayfach/IGGdb.

Field-specific reporting

Please select the one below that is the best fit for your research. If you are not sure, read the appropriate sections before making your selection.

☐ Life sciences ☐ Behavioural & social sciences ☒ Ecological, evolutionary & environmental sciences

For a reference copy of the document with all sections, see nature.com/documents/nr-reporting-summary-flat.pdf

Ecological, evolutionary & environmental sciences study design

All studies must disclose on these points even when the disclosure is negative.

Study description	Previous studies have found that a large proportion of species in the gut microbiome lack a sequenced genome. We addressed this problem by systematically recovering >60,000 draft genomes from nearly 4,000 metagenomes from phenotypically and geographically diverse human subjects.
Research sample	We downloaded 3,810 publicly available human fecal metagenome samples from the NCBI SRA spanning 15 studies.
Sampling strategy	Publicly available human gut metagenomes from major studies representing different geographic regions, lifestyles, age groups, and disease states.
Data collection	Downloaded from the NCBI sequence read archive
Timing and spatial scale	Data sets were selected to include samples from a wide range of ages (e.g. include both infants and adults), host lifestyles (e.g. urban, rural), host geography (e.g. United States, Denmark, Spain, Italy, Sweden, Finland, Estonia, Russia, Peru, El Salvador, Tanzania, Fiji, and China), and disease states (e.g. rheumatoid arthritis, diabetes, colorectal cancer, and autoimmunity)
Data exclusions	Several data sets from already well-samples regions (e.g. Europe and China) were excluded, which was pre-determined at the outset of the study.
Reproducibility	n/a
Randomization	n/a
Blinding	n/a

Did the study involve field work? ☐ Yes ☒ No

Reporting for specific materials, systems and methods

We require information from authors about some types of materials, experimental systems and methods used in many studies. Here, indicate whether each material, system or method listed is relevant to your study. If you are not sure if a list item applies to your research, read the appropriate section before selecting a response.

Materials & experimental systems

n/a	Involved in the study	
☒	☐	Antibodies
☒	☐	Eukaryotic cell lines
☒	☐	Palaeontology
☒	☐	Animals and other organisms
☒	☐	Human research participants
☒	☐	Clinical data

Methods

n/a	Involved in the study	
☒	☐	ChIP-seq
☒	☐	Flow cytometry
☒	☐	MRI-based neuroimaging
