# Supplementary material for: New insights from uncultivated genomes of the global human gut microbiome
Source: Nature. 2019 Mar 13;568(7753):505–10. doi: 10.1038/s41586-019-1058-x (PMC6784871; doi:10.1038/s41586-019-1058-x)
Supplement: Supplementary file 3 — This file contains legends for Supplementary Tables 1-20. [file 41586_2019_1058_MOESM3_ESM.pdf]

Legends for Supplementary Information

Supplementary Figure 1 | Identification and refinement of conserved OTU-specific marker genes. A) Up to 300 marker genes were identified for each of the 23,790 species-level OTUs, resulting in a total of 6,198,663 marker genes. Over 99% of marker genes are perfectly unique, with 1% found in a small percentage of other species. (Left) The box plot shows the intra-species frequency of the 6,198,663 marker genes. Values close to 100% indicate a marker gene is found in every genome of OTU. In the box plot, middle line denotes median; box denotes IQR; and whiskers denote 1.5× IQR. (Middle) Most OTUs have a full set of 300 marker genes. (Right) Human gut OTUs have fewer marker genes than OTUs from non-gut environments, which may be a result of (1) there are many closely related OTUs from the human gut, making it more challenging to identify unique markers, and (2) non-gut OTUs have fewer genomes per OTU, resulting in more marker genes found across all genomes by chance. B) Example of co-variance-based marker-gene refinement for eight human gut species. All marker genes are expected to be present in a metagenome when an OTU is present and absent when the OTU is absent. Metagenomic read-mapping was performed to identify and remove marker genes failing this condition: 3,810 human gut metagenomes were mapped to the 6,198,663 marker genes for 23,790 OTUs and 1,402 OTUs were detected in >10 samples with >1x depth and were subjected to co-variance-based refinement. The histograms show the distribution of Pearson correlation coefficients of read-depth between marker genes across samples for eight randomly chosen OTUs. The sample size of each histogram is indicated by the number of pairwise comparisons between marker genes (see panel legends).

Supplementary Figure 2. IGGsearch accurately estimates OTU presence and abundance in benchmark datasets. A) IGGsearch was applied to metagenomes from the first CAMI challenge (https://data.cami-challenge.org/participate). The low, medium, and high complexity datasets contained 60, 232, and 1,074 genomes respectively. Of these, 35, 128, and 588 could be assigned to one of the OTUs in the IGGsearch database based on a gANI value of >95%. (Left) Scatterplots of true versus estimated relative abundance for all 23,790 OTUs in the IGGsearch database. Red regression lines are from Pearson correlations. (Right) The presence-absence of all 23,790 species OTUs in the IGGsearch database was predicted at different cutoffs, defined at the % of marker genes detected per OTU. The green line indicates the true positive rate (TPR), or the fraction of OTUs present in the metagenome that were detected at a given cutoff. The red line indicates the false discovery rate (FDR), or the fraction of detected OTUs that were not present. The green line indicates the F1-score, or the harmonic mean between the TPR and FPR. The vertical dotted line at 15% indicates the cutoff we selected for prediction of OTU presence, which maximizes F1-score and indicates an optimal balance between the TPR and FDR. B) To evaluate the effect of sequencing depth we constructed mock metagenomes containing between 500,000 and 50,000,000 100-bp paired-end (PE) sequencing reads with an Illumina error model. Each metagenome contained 100 isolate reference genomes from 100 distinct OTUs that were exponentially distributed with a maximum abundance of 5%. (Left and Right) Figures follow the same format as in (A).

Supplementary Table 1. Published human gut metagenome studies used for MAG recovery. Public metagenomes were downloaded from the NCBI SRA from 15 published studies. These data represent 3,810 samples, 11,523 sequencing runs, and 181 billion reads.

Supplementary Table 2. Sequencing, assembly, and binning statistics for metagenomes. Table contains detailed assembly and binning metadata for 3,810 human gut metagenomic samples. 39 samples failed to assemble and 53 failed to bin.

Supplementary Table 3. Available human phenotypic information for metagenomic samples. Metadata for all 3,810 metagenomes used for assembly and binning, including gender, age, country, lifestyle, and BMI. Disease and other misc. information are provided in the attribute field.

Supplementary Table 4. Summary of MAG recovery by geographic region. The total number of MAGs recovered from each continent as well as average quality levels of recovered MAGs.

Supplementary Table 5. Quality information for 60,664 MAGs from the HGM dataset. Table includes CheckM estimates of completeness and contamination, assembly statistics, read-depth, counts of tRNA and rRNA genes, and SNP density.

Supplementary Table 6. Application of MAGpurify to HGM dataset. Columns A-C describe the strategies used to identify and remove contamination from MAGs. Columns D-H report the results of each strategy after application to the entire HGM dataset, including low quality bins. A total of 5.2 million contigs were removed, representing 7.1% of the total number of binned contigs.

Supplementary Table 7. Evaluation of MAGpurify using 1000 simulated MAGs. Host name indicated the genome name of the dominant organism in each MAG, and donor name the genome of the contaminating organism**.** Host and donor organisms are distinct from each other at the species level, high quality genomes, and all isolated from the human gut
True completeness is defined as the fraction (i.e. copy number) of the host genome present
True contamination is defined as the fraction (i.e. copy number) of the donor genome present
Distributions of N50, completeness, and contamination are based on the HGM dataset.

Supplementary Table 8. Evaluating MAGs using previously published quality standards. The MAGs from the HGM dataset were subjected to several previously published quality standards, including that of Bowers et al. 2017, Parks et al. 2017, and the HMP.

Supplementary Table 9. Information for 153,900 total non-redundant reference genomes. Reference genomes were obtained from PATRIC & IMG. 145,917 genomes meet the medium quality MIMAG standard and were retained for further analysis. All genomes were made non-redundant based on Mash clustering at a distance threshold of 0.0. Clusters of redundant genomes are indicated by the 'clustered_genomes' field.

Supplementary Table 10. Metadata for all 23790 species in the IGGdb, including 4558 gut species and 2058 new species. Isolation source is based on metadata from GOLD and PATRIC. When multiple sources were indicated, preference was given in the following order: human gut, human, host-associated, non-host-associated**.** Gut species were defined based on (1) isolation metadata, (2) presence of a HGM, or (3) metagenomic read mapping with IGGsearch**.** The representative genome was chosen to maximize genome quality and relatedness to other strains of the same species

Supplementary Table 11. Mapping between CAMI genomes to IGGdb species. All 1366 genomes, plasmids, and viruses from CAMI were assigned to a species from the IGGdb based on >=95% genome-wide average nucleotide identity (ANI): 752/754 Bacterial and Archaeal genomes from CAMI were assigned to an IGGdb species; 0/19 Viruses from CAMI were assigned to an IGGdb species; 0/559 Plasmids from CAMI were assigned to an IGGdb species; 14/34 unannotated CAMI genomes were assigned to an IGGdb species.

Supplementary Table 12. IGGsearch predictions of CAMI datasets. All three CAMI dataset were run using IGGsearch to predict species presence and estimate species abundance. A species was predicted as present if at least 15% of marker genes recruited at least 1 read. Each species was classified as a True Positive (TP), False Positive (FP), True Negative (TN) or False Negative (FN): TP = 557; FP = 2; TN = 70811; FN = 0. The number of true negatives accounts for species absent from CAMI that were not reported by IGGsearch due to 0 mapped reads.

Supplementary Table 13. Relative abundance and richness of new species across studies. Table indicates the distribution of new species across four datasets used for MAG recovery as well as six additional MAG datasets not used for MAG recovery.

Supplementary Table 14. Correlations of new species with community diversity for individual studies. Species abundance was estimated using IGGsearch after rarefying each metagenome to 1M reads. Community diversity was measured using either species richness or Shannon entropy of species relative abundances. New species were measured as the % of total richness or the % of total abundance. Correlations were performed using Pearson correlation.

Supplementary Table 15. Metagenomic samples used for identifying species-disease associations. To prevent confounding signals due to disease treatment, we excluded 100 individuals taking drugs that affect microbiome composition, including metformin in T2D patients, acarbose, atorvastatin, fondaparinux, and metoprolol in ACVD patients, and antirheumatic drugs in rheumatoid arthritis patients.

Supplementary Table 16. P-values from association of species abundance with disease. IGGsearch, MIDAS, mOTU, and MetaPhlAn 2 were used to estimate the abundance of species across 10 metagenome studies of disease. Species names and taxonomy are only reported for IGGsearch species. For IGGsearch, new species are indicated by names containing the prefix HGM. P-values are from two-sided Wilcox rank sum tests of species abundance between cases and controls. P-values were corrected for multiple hypothesis tests per disease using the FDR procedure. Only corrected p-values (q-values) <0.10 are reported in table.

Supplementary Table 17. Listing of OTUs used for comparative genomics between cultured and uncultured bacteria. Cultured species-level OTUs contain at least one genome from a cultivated isolate, while uncultured OTUs are represented exclusively by MAGs from the HGM dataset or previous studies. Prevalence is based on IGGsearch profiling and defined as the % of metagenomes from healthy adults where the species-level OTU was detected. Only OTUs found in at least 5% of metagenomes were included in the analysis.

Supplementary Table 18. Genomic differences between cultivated and uncultivated human gut bacteria. Five features were compared between 271 uncultivated and 233

cultivated species-level OTUs using a two-sided Wilcoxon rank-sum test. Genomic features were averaged across all MAGs per OTU. The tested genomic featured included: 1) observed genome length: average observed genome length across all high quality MAGs; 2) estimated genome length: this is corrected for estimated % completeness and contamination in each MAG; 3) coding density: percent of observed length that falls into protein coding regions; 4) GC content: percent of observed length comprised of G + C nucleotides; 5) growth rate: estimated using the iRep tool.

Supplementary Table 19. Differences in KEGG functional groups between genomes of cultured and uncultured species. Functions from the KEGG database were compared between high-quality MAGs from cultivated and uncultivated human gut species-level OTUs. The presence-absence of KEGG functions were averaged across all MAGs per species-level OTU. The Ives-Garland method, implement in the phylolm package, was used to associate genes with culture status while controlling for phylogeny.

Supplementary Table 20. ENA accessions for representative MAGs from 2058 new species OTUs. These MAGs have been deposited into the ENA under study accession PRJEB31003.
